# 

**Published:** 1895-02-23

**Authors:** 


### CONTENTS.

**Isolation Hospitals as Centres of Infection** - - - 359
**Treatment and the Pulmonary Circulation.** By Sir B. W. Richardson, M.D., F.R.S. IV. - - - 361
**Modern Sociology.**—The Price of the Publican - - - - 362
**Notes and News** - - - - - - - 364
**Medical Progress and Hospital Clinics:**
On Some Minor Anal and Rectal Ailments.—I. - - - - 365
Progress in Surgery.—Hernia - - - - - - 366
Progress in Dermatology - - - - - - 367
Therapeutic Notes - - - - - - - 370
New Appliances and Things Medical - - - - - 370
**Editor's Letter Box** - - - - - - - 370
**Annotations.**—Wholeman Specialism — Medical Officers and Public Hospitals—The Pay System at the Great Northern Central 363
**The Institutional Workshop:**
Hospital Finance.—Special Hospitals: Robbers of the Poor - 371
Hospital Administration. — Paying Patients at the Great Northern Central Hospital - - - - - - - 372
Practical Departments.—The Hospital Locker - - - 372
Hospital Festivals and Meetings - - - - - - 373
**Scraps and Gleanings** - - - - - - - - - 374
**The Book World of Medicine and Science** - - - - 375
**The Student of Medicine** - - - - - - - - 375

### EXTRA SUPPLEMENT.—THE NURSING MIRROR.

**News from the Nursing World.**—Creating a Grievance—Miss Gibson's Paper—Who Should Inspect?—East Grinstead Cottage Hospital—District Nurses at Birmingham—Nurses at Lichfield—The Trained Nurse at Ely—Shepton Mallet—The Question of Religion—Kind, but Unskilled—Kilmarnock Infirmary—Charity in Dublin—The President at Belleville—A Pleasant Evening—Carols at Sydney—Short Items - - - - cliii

**Presentations** - - - - - - - - - clv

**Royal British Nurses Association** - - - - - clvi

**Elementary Anatomy and Surgery for Nurses.**—VII. The Principal Joints of the Body; with the Chief Muscles which move them - - - - - - - - - clv

**Lewisham Infirmary** - - - - - - - - clvi

**English Nurses at Hong Kong** - - - - - - clvi

**Appointments** - - - - - - - - - - clvi

**Burdett's Official Nursing Directory** - - - - clvii

**Legal Intelligence** - - - - - - - - - clvii

**The Meath Hospital and County Dublin Infirmary** - clviii

